# Eco-Friendly Sol–Gel Coatings with Organic Corrosion Inhibitors for Lightweight AZ61 Alloy

**DOI:** 10.3390/gels10030168

**Published:** 2024-02-27

**Authors:** Jorge Domínguez-Martínez, Jesús López-Sánchez, Federico García-Galván, Aída Serrano, Violeta Barranco, Juan Carlos Galván, Óscar Rodríguez de la Fuente, Noemí Carmona

**Affiliations:** 1 Department of Materials Physics, Universidad Complutense de Madrid (UCM), 28040 Madrid, Spain; jordom02@ucm.es (J.D.-M.); osrodrig@ucm.es (Ó.R.d.l.F.); 2 Department of Electroceramics, Instituto de Cerámica y Vidrio (ICV-CSIC), 28049 Madrid, Spain; jesus.lopez@csic.es (J.L.-S.); aida.serrano@icv.csic.es (A.S.); 3 Department of Engineering, School of Architecture, Engineering and Design, Universidad Europea de Madrid, 28670 Villaviciosa de Odón, Spain; federico.garcia@universidadeuropea.es; 4 National Center for Metallurgical Research (CENIM-CSIC), Avda. Gregorio del Amo 8, 28040 Madrid, Spain; violeta.barranco@csic.es (V.B.); jcgalvan@cenim.csic.es (J.C.G.); 5 Applied Magnetism Institute (IMA) “Salvador Velayos”, A6 Km 22,5, Las Rozas, 28230 Madrid, Spain

**Keywords:** AZ61 alloy, sol–gel, protective coating, corrosion eco-inhibitors

## Abstract

The latest advances in technology and materials science have catalyzed a transformative shift towards the adoption of environmentally conscious and lightweight materials across key sectors such as aeronautics, biomedical, and automotive industries. Noteworthy among these innovations are the magnesium-aluminum (Mg-Al) alloys employed in aeronautical applications, contributing to the overall reduction in aircraft weight and subsequently diminishing fuel consumption and mitigating atmospheric emissions. The present work delves into a study of the anti-corrosive properties inherent in various sol-gel coatings, leveraging a range of environmentally friendly corrosion inhibitors, specifically tailored for samples of the AZ61 alloy. Methodologically, the work involves the synthesis and application of sol-gel coatings on AZ61 alloy containing eco-friendly inhibitors: L-cysteine, N-acetyl-cysteine, curcumin and methylene blue. Subsequently, an accelerated corrosion test in a simulated saline environment is performed. Through microstructural and compositional analyses, the best inhibitors responses are achieved with inhibitors containing S, N heteroatoms and conjugated double bonds in their structure, probably due to the creation of a continuous MgCl_2_ layer. This research contributes to the ongoing discourse on protective eco-coatings, aligning with the broader paradigm shift towards sustainable and lightweight materials in key industries.

## 1. Introduction

In the dynamic field of the aeronautical industry, innovation is a constant driving force for progress. The industry consistently strives to redefine technological and design boundaries, as evidenced by the ongoing development of cutting-edge prototypes. Integral to this mission is the meticulous selection and understanding of optimal materials for aircraft and vehicle designs. The consequences of any oversight at the structural or material level are profound, with the potential for catastrophic outcomes [1]. 

Currently, there is a pressing need for aircraft materials to maintain optimal lightweight properties, a crucial factor in the ongoing efforts to minimize aircraft weight and, consequently, decrease emissions and environmental pollutants. In response to this imperative, various alternatives are under scrutiny, with magnesium alloys emerging as a prominent focus among numerous options. These alloys present the potential to be utilized either independently or in conjunction with other lightweight materials, forming composite structures that combine strength and efficiency in aerospace applications [2]. These lightweight alloys, exemplified by magnesium alloys, exhibit commendable strength, ductility, and lower densities in contrast to materials like titanium or aluminum alloys. While it is acknowledged that their malleability is relatively lower than that of aluminum, the advantage lies in the ability to attain lower temperatures and forces during machining, contributing to an extended service life of the material. Additionally, the exceptional recyclability of these alloys further underscores their environmental efficacy [3].

Nevertheless, the utilization of these alloys introduces another critical consideration—the potential occurrence of corrosion phenomena resulting from their interaction with humid environments. Furthermore, factors such as grain size, dislocation density, and texture emerge as crucial parameters that can significantly influence the corrosion resistance of these alloys [4,5,6]. Hence, to mitigate the potential corrosion risks, it becomes imperative to employ surface treatments or apply protective coatings. Common approaches involve the use of paints or employing anodizing processes. Notably, the sol–gel process emerges as one viable option, offering economic efficiency and versatility. Its cost-effectiveness makes it a preferred choice, while its adaptability allows for application across a spectrum of alloy types. Furthermore, it is noteworthy that the development of the sol–gel process has experienced substantial growth over the years, underscoring its evolving significance in corrosion protection methodologies [7].

The sol–gel process comprises three distinct stages: hydrolysis, polycondensation, and thermal densification. It initiates in the sol phase, characterized by a colloidal suspension of small particles in a solution, and subsequently progresses into the gel phase, exhibiting a solid–liquid biphasic structure. An advantageous aspect of the sol–gel process lies in the flexibility of the precursor materials, which can be either organic or inorganic, distinguishing it from other methods. Noteworthy attributes of the sol–gel process include its economic viability, minimal toxicity, and the high purity of the resultant products achieved through meticulous control of the precursors’ composition. The coatings generated exhibit commendable chemical resistance, functioning as potent corrosion inhibitors for both metals and alloys. Moreover, the synthesis can be conducted at room temperature and atmospheric pressure [8,9], adding to its practicality. The distinctive nanoporosity of sol–gel coatings introduces an additional dimension to their functionality. This feature allows them to actively combat corrosion by harboring new molecules while preserving their initial properties, paving the way for innovative applications in diverse fields [10,11].

In the pursuit of enhancing corrosion protection in coatings, the incorporation of inhibitors offers a strategic approach. These inhibitors play a dual role by either retarding the degradation of alloys, forming a barrier against corrosion, or engaging in a self-repairing mechanism for active protection. In the wake of the ban on chromate treatments, various inorganic salts, including Ce^3+^, Zr^3+^, or La^3+^ ions, have historically served as inhibitors. However, a contemporary shift toward organic molecules and their derivatives is evident, driven by their superior biodegradability and reduced toxicity and environmental impact [12]. The recent transition to organic inhibitors is motivated by their enhanced inhibitory capacity, attributed to the presence of multiple heteroatoms and double bonds. This shift reflects a commitment to advancing corrosion protection methods in a more sustainable and environmentally conscious direction [13,14,15,16,17,18]. 

The present work is dedicated to the application and characterization of diverse sol–gel coatings utilizing environmentally friendly corrosion inhibitors. These coatings will be applied to different samples of AZ61 alloy, specifically targeting applications in sustainable transport and determining their anticorrosive capacity. The overarching objective is to contribute to the advancement of environmentally conscious coatings for the promotion of sustainability in transportation.

## 2. Results and Discussion

### 2.1. Coatings Characterization

Table 1 displays the obtained coatings with their corresponding nomenclature, preparation, and test conditions.

The thickness of the coatings was determined through the interference of reflection spectra in the ultraviolet-visible and near-infrared ranges [19]. Specific values for each sol–gel coating are presented in Table 2. The approximate thickness of all coatings ranged between 150 and 300 nm.

**Figure 1:**
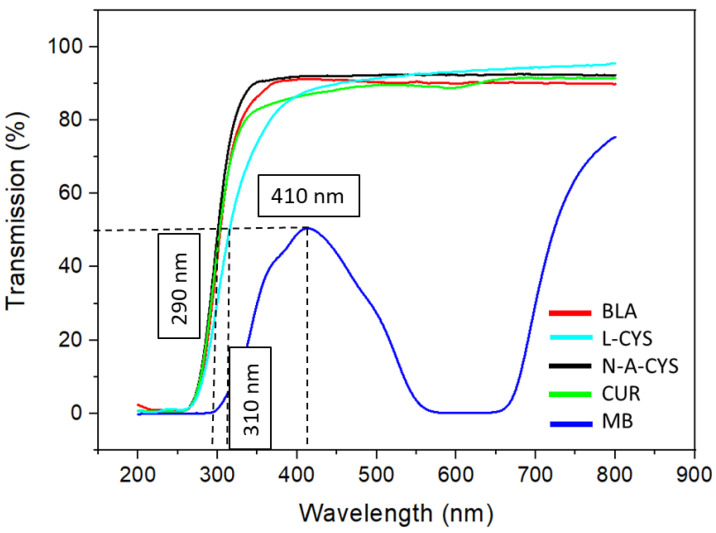
Transmission spectra related to N-A-CYS, BLA, CUR, MB, and L-CYS sol–gel coatings.

The transmission spectrum of the coated glass samples is performed in order to determine the wavelength of each coating at 50% transmission. For this purpose, a line is drawn parallel to the X-axis from the 50% transmission point until the cut-off with the corresponding curve occurs. After this, a line is drawn perpendicular to the X-axis up to this point, thus obtaining the corresponding wavelength values. As depicted in Figure 1, the sample containing the MB inhibitor exhibits the closest to the visible wavelength value, approximately at 410 nm. This suggests strong transmission within this segment of the visible spectrum, with absorption occurring in the range of 600–650 nm, resulting in a blue appearance. Following this, the sample with the L-CYS inhibitor shows an absorption edge wavelength around 310 nm. Finally, the last three samples (BLA, N-A-CYS, CUR) demonstrate a wavelength very close to 50% transmission, with values around 290 nm, highlighting the high transparency of the coatings within the visible spectrum and part of the near UV range.

**Figure 2:**
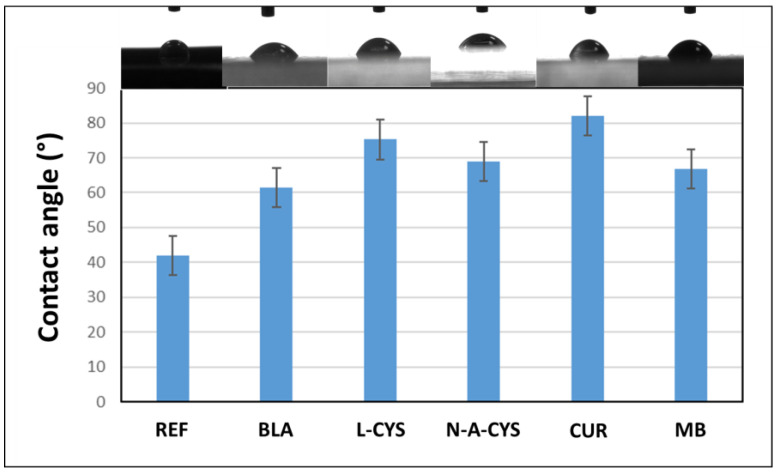
Contact angle values obtained for the samples REF, BLA, L-CYS, N-A-CYS, CUR, and MB with distilled water. Images of the water droplets on the surfaces on top of each sample.

To determine the hydrophilic character of the coatings, the samples are characterized by contact angle measurements before the corrosion test. The procedure consists of depositing a drop of water on the coating surface and illuminating it with diffuse light in order to project the image through a camera onto a computer screen, thanks to which the tangent of the angle formed by the drop of water with the surface of each coating can be measured. In this work, different measurements were made using 1 μL drops of distilled water deposited on the surface of the coatings. Three different measurements along the surface of the sample were made to average the final result. Figure 2 lists the final contact angle values for each sample along with their respective uncertainty. 

As is evident from the results, all coatings exhibit a hydrophilic nature, as indicated by contact angles less than 90°. These hydrophilic properties of the coatings have been shown to be stable with reproducible contact angle values maintained for several months. The L-CYS and CUR coatings demonstrate the most favorable outcomes due to their values closely approaching 90°, suggesting their suitability for practical coating applications. By contrast, the REF sample, lacking any coating, stands out as expected, showing a relatively low contact angle compared to the others. Following closely is the BLA sample. This implies that the surfaces with lower contact angles, such as those of REF and BLA, are more prone to retaining water droplets, as the area in contact with water is greater than surfaces with higher contact angle values. 

### 2.2. Weathering Test

After the thermal treatment of the AZ61 alloy samples for their densification, the study of their corrosion behavior in a concentrated saline medium is started. For this purpose, a variation of the ISO 11130 standard was used [20]. Each sample is attached with a fishing line and immersed in a 0.6 M NaCl solution in 125 mL Teflon bottles without stirring for 14 days. The solution temperature is 25 ± 2 °C and the solution was not changed during the test. Once the test is finished, the samples were washed with distilled water by immersion in three different beakers to ensure that they are properly cleaned of residues and/or impurities. Afterwards, the samples were dried at 25 ± 2 °C in a desiccator at less than 10% relative humidity for a further 14 days. The pH for each salt solution was measured before and after the test (including one with distilled H_2_O + NaCl, with initial and final pH = 7) obtaining values of pH = 5, except in the samples with the L-CYS and MB coating where it is pH = 4. This indicates that protons are released into the saline medium causing an acidification of the latter during the corrosion process, leading, in all probability, to the following reaction:

↓ Mg + 2 H_2_O→ ↓ Mg(OH)_2_ + 2H^+^ (1)

**Figure 3:**
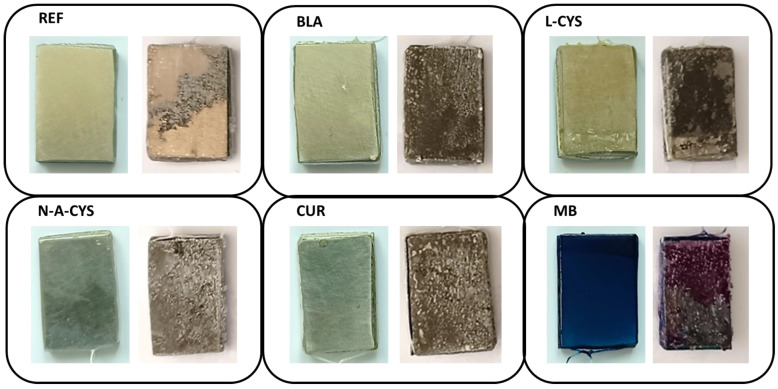
Photographs of reference and samples with sol–gel coatings before corrosion test (left image) and after corrosion test (right image).

The different samples before and after the corrosion test are shown in Figure 3, where clear differences can be appreciated.

It can be observed that all the samples show a superficial deterioration after 2 weeks immersed in saline medium. White deposits of crystalline appearance can also be seen in all of them, indicating the presence of corrosion products on their surface. The sample with the MB coating is the one in the poorest condition since its coating layer has partially detached.

### 2.3. Optical Microscopy Characterization 

**Figure 4:**
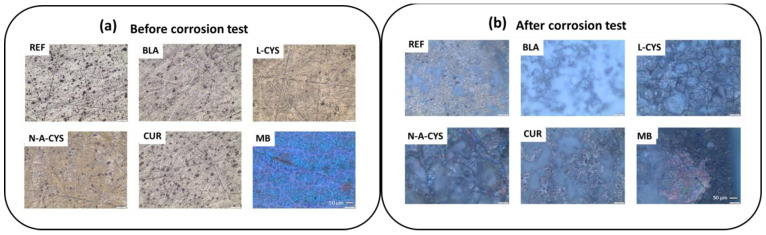
Optical micrographs of the samples: (**a**) before; and (**b**) after the corrosion test.

Optical microscopy provides information about the surface of the samples both before and after the corrosion test. Interestingly, significant structural changes occur in each sample as identified in Figure 3. Figure 4 gives an overview of the micrographs obtained where the modifications at the microstructural level are shown.

Figure 4a shows the surface of the different samples initially. The traces of the polishing prior to the application of the coating can be seen, which is carried out to remove any remaining oxide or dirt, but additionally the process improves the adhesion of the coating to the alloy surface. After the corrosion test in Figure 4b, the surface of all samples shows white crystalline deposits. Besides, in sample MB, the lifted coating is visible as can be observed in its respective micrograph by contrasting the brightness of each zone. In the L-CYS sample, cracks generated by the etching of the grain boundaries leading to grain detachment are observed along the entire surface.

### 2.4. Weight Variation

The samples were weighed before and after the corrosion test to examine weight variations. For accuracy, the samples were washed with distilled water after the test and left for 1 week in a desiccator to remove the humidity caused by the washing.

**Figure 5:**
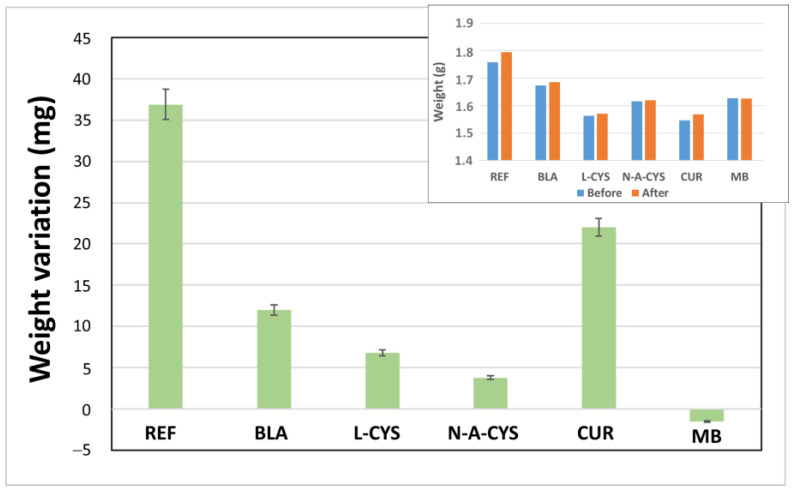
Weight variation of the samples before and after corrosion test. Inset shows absolute values of the weight before and after the test.

As a general remark, relatively high differences are noticed in the weight of the samples both before and after the corrosion test (Figure 5). In the MB sample, a negative weight variation of −1.5 mg is observed, which could be explained by a partial detachment of the sol–gel coating from the metal surface. In turn, the metal surface reacts with the ions of the medium, causing its dissolution. However, for the rest of the coated samples, the weight is slightly increased, probably due to the formation of corrosion products in the form of metal oxides, hydroxides or chlorides on the surface when the metal ions themselves react with the OH- ions of the saline medium. The uncoated sample (REF) has an excess weight of more than 35 mg (36.9 mg) and the CUR sample displays an increase of more than 20 mg (22.0 mg). In a second group, we can find the samples L-CYS and BLA showing an increase between 7 and 11 mg and finally the sample N-A-CYS, which barely modifies its weight (3.8 mg) (Figure 5).

Results show how a thin layer of a sol–gel coating of less than 300 nm (BLA) is sufficient to significantly reduce corrosion on the alloy surface without coating (REF). Although it is logical to think that the thicker the coating is, the greater is its protective effect on the alloy, it is not always the case. Sol–gel coatings have an inherent nanoporosity in their structure and do not exert a barrier effect like other organic polymers (i.e., epoxy resins or polyurethanes). In the sol–gel coatings including organic inhibitors, the corrosion inhibiting effect of the chosen molecules must also be taken into account. Some previous studies mention that organic compounds bearing heteroatoms with high electron density such as phosphor, sulphur, nitrogen, oxygen or those containing multiple bonds are considered as adsorption centers, and thus are effective as corrosion inhibitors [21]. Compounds containing both nitrogen and sulphur in their molecular structure have exhibited greater inhibition compared with those containing only one of these atoms. The inhibitor seems to be chemically or physically adsorbed via both the thiol S atom and the carbonyl O atom to the metal and thus results in a higher experimental inhibition efficiency [22]. The non-bonding electron pair of N, O and S are known to adsorb onto metallic surfaces by electrostatic and electronic interactions with the vacant d orbital of iron. Due to these interactions, they can create a barrier layer on the metal, which displaces water and blocks the attack of aggressive species [23]. It seems like the sol–gel coating thickness improves the corrosion resistance as a barrier effect and the addition of organic inhibitor molecules improves the anti-corrosion effect.

### 2.5. SEM Characterization

**Figure 6:**
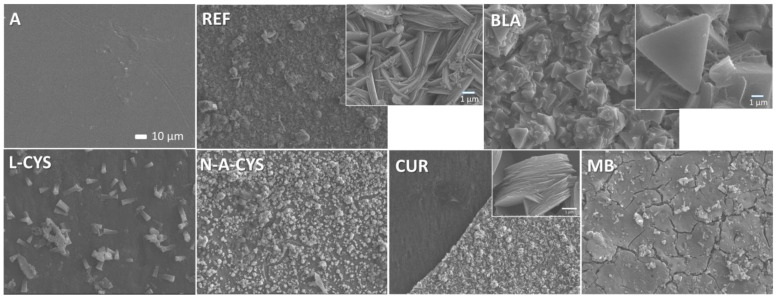
Representative SEM images of the surface of samples A, REF, BLA, L-CYS, MB, CUR, and N-A-CYS.

The microstructure and surface morphologies were investigated by scanning electron microscopy (SEM), as in Figure 6. In addition, energy dispersive X-ray (EDX) analyses were carried out to reveal the compositions of the materials formed, which are listed in Table 3. The first sample analyzed is sample A, which corresponds to the AZ61 alloy without a coating or corrosion test. The SEM image shows a clean surface, with no traces of corrosion products or impurities. EDX analyses of the surface area of the image in Figure 6 A show the occurrence of Mg (92.5 wt.%) and Al (7.5 wt.%). Therefore, it indicates that no oxidation occurs on the surface. Regarding the REF sample (uncoated but submitted to the corrosion test), it is possible to observe flake-shaped crystals and certain grains with a rounded shape and more whitish color. The chemical analysis of the grains in the inset of Figure 6 REF shows a composition with Mg (66.7 wt.%), some Al (3.7 wt.%) and O (29.7 wt.%). Interestingly, the REF sample has a high amount of O, suggesting that it does possess a high amount of corrosion products on its surface. As for the whitish grains found in this sample, a spot analysis shows that they have a lower amount of O, implying that they are not as strongly oxidized as the background of the sample. Finally, the flaky crystals show a similar O composition to the background, so they also experienced a high oxidation. The BLA sample indicates a composition with Mg (39.5 wt.%), O (57.8 wt.%) and a small amount of Cl (2.7 wt.%) in its surface analysis. The high concentration of O along with Cl, is probably due to remnants of the NaCl solution that have not been washed out after the post-test wash. In turn, triangular-shaped crystals composed by different layers display a similar composition. As the percentage of Cl is residual, the formation of Mg hydroxide is probable.

In the L-CYS sample, surface area analysis reveals a predominant presence of Mg (77.0 wt.%) and O (23.0 wt.%), suggesting a relatively low concentration of O and indicative of less aggressive corrosion. Additionally, a smooth surface featuring small whitish rods is observed, with a rod composition made by a spot analysis comprising Mg (46.1 wt.%), O (51.7 wt.%), and a minor proportion of Al (2.3 wt.%). The structural background analysis reveals the presence of Si (1.2 wt.%), originating from the sol–gel coating. The surface composition analysis of the MB sample reveals a composition of Mg (50.1 wt.%), O (38.1 wt.%), Al (2.2 wt.%), and Si (9.6 wt.%). The presence of cracks on the surface indicates that the coating persists and, as in the L-CYS sample, a small proportion of Si was also detected. While the observed O content exceeds that of the L-CYS sample, it does not reach the levels identified in the REF sample; instead, it falls within an intermediate range.

Interestingly, two analyses are conducted on the CUR sample: one on the smoother and darker surface (left area), and the other one on the so-called crust zone (lighter right area). In the left area, C (19.2 wt.%) and Al (2.8 wt.%) appear together with O (17.7 wt.%) and Mg (60.3 wt.%), while in the lighter right area, there are only Mg (43.0 wt.%) and O (57.0 wt.%). In the separation region between the two areas (interface) can be seen rounded flaky crystals in the crust area. Moreover, a large flake-like crystallization is visible. The sample N-A-CYS presents all types of the constituents after surface analysis. It contains Mg (43.1 wt.%), O (43.1 wt.%), Al (1.1 wt.%), C (9.0 wt.%), Si (2.7 wt.%) and Cl (1.0 wt.%). Cracks were also found, indicating that the coating is present on the surface together with small triangular-shaped crystals with a different contrast. In the CUR sample, the crust area analysis displays the presence of O and Mg, suggesting the deposition of the corresponding hydroxide. In the smooth area, low values of O and Al are observed, accompanied by some C (19.2% by weight), possibly attributed to the thin layer of organic–inorganic coating. Finally, the N-A-CYS sample exhibits the presence of all the aforementioned elements. The concentration of Si indicates the presence of the coating, the percentage of Cl suggests minimal traces of marine dissolution, and the ratio of O to Mg indicates that the oxidation is not overly abrupt. 

**Figure 7:**
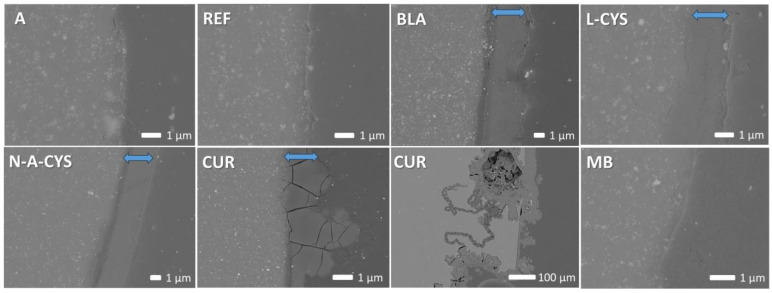
Representative SEM images from the cross-section of the samples A (non-corroded), REF, BLA, L-CYS, MB, CUR, and N-A-CYS (after corrosion test). The double blue arrow indicates the coating together with the corrosion products formed after the corrosion test.

The SEM images from the cross-section of the samples in Figure 7 show sharp interfaces corresponding to the alloy (on the left side of the image) and the right side, corresponding to the epoxy resin of the embedding material for the non-corroded samples A and REF. The coatings of samples BLA, L-CYS, N-A-CYS and CUR, together with the corrosion products formed after the corrosion test, are marked in the SEM images for each of them with a blue double arrow. Their thicknesses are around ~1–2 μm and their composition indicates the presence of a large amount of Mg (40.0 wt.%), O (58.6 wt.%) and a small amount of Si (1.4 wt.%). The initial thickness of the coating seems to have increased due to oxidation of the magnesium at the alloy-coating interface. The adhesion of the coatings is, however, good due to the formation of Si-O-Mg chemical bonds and therefore no flaking is observed.

In the SEM image of the CUR sample, the swollen coating is completely cracked, and in a lower magnification image, corrosion penetration lines can be seen, while the layer is peeling off in some areas. Finally, in the MB sample, an irregular interface can be seen where no coating or corrosion layer is visible. This is in agreement with the superficial analysis performed, which showed that the coating had peeled off in a large part of this sample. In contrast to the surface SEM images (Figure 6), no crystals are visible from the cross-section in Figure 7, which have probably been dissolved during polishing.

### 2.6. Confocal Raman Microscopy Characterization

**Figure 8:**
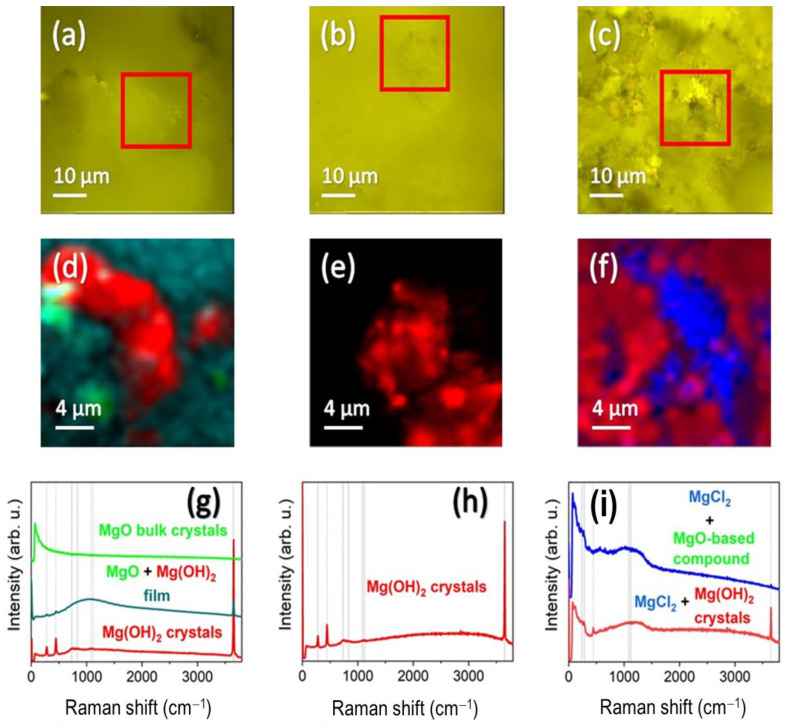
(**a**–**c**) Optical images (red squares indicate the representative areas where Raman mapping was performed); (**d**–**f**) superficial integrated Raman intensity images, (**g**–**i**) averaged Raman spectra obtained for the REF, BLA, and N-A-CYS samples.

Confocal Raman microscopy is a very versatile technique, and it can be highly useful in this type of corrosion study since it provides accurate compositional information at a local microscale level [24,25,26,27]. Specifically to this study, it enables us to know about the compounds formed considering the chemical strategies developed in the protective sol–gel coating. These compounds formed could hold the key to understanding the ability to withstand corrosion, in this case, in a saline environment. For this purpose, an uncoated (REF), coated (BLA) and coated with one type of inhibitor sample (N-A-CYS) were chosen. Optical images corresponding to representative zones are displayed in Figure 8a–c, respectively. The REF and BLA samples exhibit large homogeneous flat areas and no particle aggregates or evidence of a deposited coating on the BLA sample, as shown in the SEM images in Figure 6. In contrast, the N-A-CYS sample exhibits heterogeneity in its microstructure, alternating both flatter zones and particle aggregates, thus evidencing a high degree of surface roughness. In addition, there are easily found areas that form optical iridescence, characteristic of a silica-based sol–gel coating [28]. 

Raman mappings were performed on representative areas indicated with a red square in Figure 8a–c and different Mg-based compounds were identified. The integration of their main Raman bands was carried out and the in-plane Raman image was calculated (Figure 8d–f). Subsequently, the average Raman spectra of the compounds formed are retrieved and plotted in Figure 8g–i. Starting with the REF sample (Figure 8d,g), we notice the formation of bulk crystals of MgO with no contribution of hydroxyl groups (green color), agglomerates of Mg(OH)_2_ nanocrystals (red color), and a continuous layer composed mainly of MgO and Mg(OH)_2_ (turquoise color). The Raman spectrum of MgO is very characteristic since its rocksalt structure means that Raman modes are forbidden for bulk crystals [29]. Therefore, we are dealing with a flat Raman spectrum without observable Raman mode features.

It is true that this Raman spectrum could properly belong to the AZ61 metal alloy itself. However, given the high weight gain (Figure 5) and the high degree of oxidation detected (Table 3), we consider that the alloy surface has mostly oxidized after the corrosion test. Interestingly, several main Raman modes at 280, 446, 1088, and 1120 cm^−1^ are reported when the particle size is between 30 and 3000 nm [30,31]. Such Raman modes are clearly identified in the Raman spectrum obtained in the REF sample and they are indicated by dotted lines. Further modes also appear within the first-order Raman scattering such as the one around 730 cm^−1^ [31]. It is worth mentioning that the 1088 and 1120 cm^−1^ modes are fairly wide, spanning a long range between 800 and 1500 cm^−1^. They correspond to surface phonon modes within the TO-LO phonon gap [29]. In contrast, Mg(OH)_2_ shows great similarities with respect to MgO, except for the band located around 3600 cm^−1^, corresponding to OH bonds [32]. Moreover, the formation of Mg hydroxides is very likely since MgO is highly hygroscopic and interacts with physisorbed water to form Mg(OH)_2_ [33]. 

**Figure 9:**
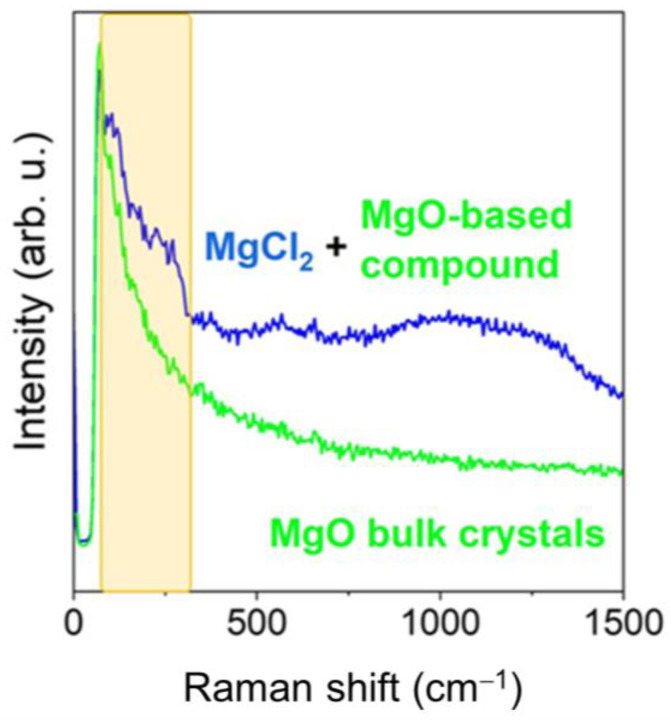
Raman comparison between MgO bulk crystals and MgCl_2_ + MgO-based compound.

The BLA sample (with coating and no inhibitor), is compositionally homogeneous and the formation of Mg(OH)_2_ is noted both in the flat zones and in the agglomerates detected (Figure 8e,h). However, the N-A-CYS sample with the inhibitor shows an additional formation of MgCl_2_ with no presence of hydroxyl groups (blue color in Figure 8f,i). The presence of MgCl_2_ is predominant in all regions studied and appears mixed with Mg(OH)_2_ (red color in Figure 8f), although it is also observed mixed with a non-indexed compound, probably based on MgO. We assume that the blue Raman spectrum is an MgO-based compound since it possesses the same Raman characteristics as MgO, but the Raman bands are shifted towards lower wavenumbers. In addition, this non-indexed compound shows a low crystallinity as the Raman bandwidth is wide and the relative intensity of the Raman modes is really low. Coming back to the Raman features of MgCl_2_, this compound features its Raman allowed modes below 300 cm^−1^ and can be identified mainly by the mode located around 254 cm^−1^ indicated with an X in Figure 8i [34]. For clarity, we compare the Raman spectra of MgO bulk with the mixture with MgCl_2_ in Figure 9, indicating with a yellow color the Raman spectral zone characteristic of MgCl_2_ [34,35].

## 3. Conclusions

The investigation focused on the corrosion behavior of eco-friendly sol–gel coatings on the AZ61 (Mg-Al) alloy reveals valuable insights. Notably, two distinct groups based on coating thickness emerged: one with thicknesses below 200 nm (BLA and CUR) and another with thicknesses close to 300 nm (L-CYS, N-A-CYS, and MB).

The consistent composition of the coatings, coupled with simultaneous fabrication, indicated that samples containing organic inhibitors exhibit greater thickness. This observation suggests that the inhibitors play a catalytic role in accelerating polycondensation, facilitating their adhesion to the alloy surfaces. Noteworthy variations in contact angle values, with the CUR sample approaching the 90⁰ threshold, underscored the predominantly hydrophilic character of all coatings. Through a comprehensive corrosion test conducted in a concentrated saline medium, the study aimed to assess the impact of different inhibitors on the alloy’s corrosion resistance. Weight variation assessments highlighted the pivotal role of coatings in corrosion prevention. In this sense, all samples except MB increase in weight, indicating that the coating is the key in preventing or slowing down corrosion by the formation of hydroxides and chlorides that could adhere to the surface of the samples. Intriguingly, thicker coatings (L-CYS and N-A-CYS) demonstrated superior corrosion resistance.

Comparative EDX analyses consistently reveal lower corrosion in the presence of coatings, a trend aligned with SEM image observations. This leads to the inference that organic inhibitors, particularly those with S, N heteroatoms, and conjugated double bonds, stand out as promising candidates for effectively retarding and inhibiting corrosion in light Mg-Al alloys. Raman experiments corroborate the oxidation and superficial modifications in coatings identifying several Mg-based compounds. 

Notably, the environmentally friendly characteristics of these inhibitors enhance their potential for widespread industrial use inside sol–gel coatings on Mg alloys, making them economically viable. These coatings are easily applied by dip-coating or spray, since their densification is carried out at room temperature. Their properties are maintained over time, so that a possible industrial scale-up would be possible and easily implementable. Consequently, this research not only contributes to refining corrosion mitigation strategies for such alloys but also paves the way for future developments across diverse sectors and applications.

## 4. Materials and Methods

### 4.1. AZ61 Alloy

A light magnesium–aluminum alloy, commercially known as AZ61 (0.3% by mass of Mn, 0.7% by mass of Zn, 6% by mass of Al, and the remaining portion of Mg up to 100% by mass) was used as a substrate for the samples. Subsequently, samples with dimensions 2.0 × 1.4 × 0.3 cm were polished to eliminate any traces of oxide layers and/or surface impurities that may have been present prior to the application of the coating.

### 4.2. Synthesis of the Sol–Gel Coatings for Inhibitory Performances 

**Figure 10:**
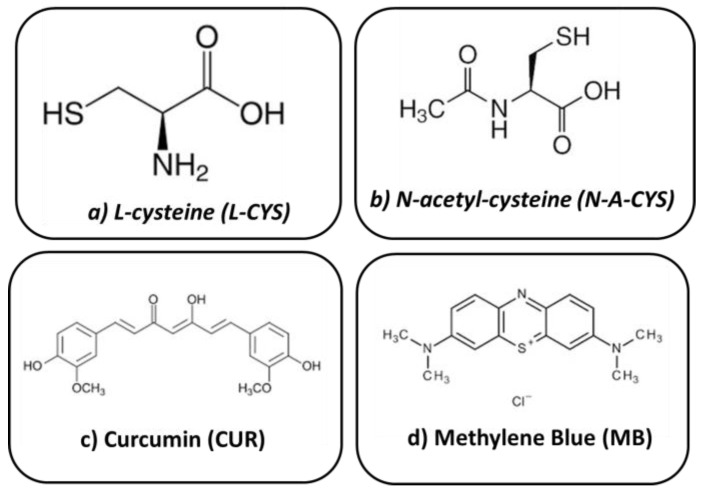
Chemical structure of corrosion inhibitors added to the sol–gel coating: (**a**) L-cysteine, (**b**) N-acetyl-cysteine, (**c**) curcumin and (**d**) methylene blue.

**Figure 11:**
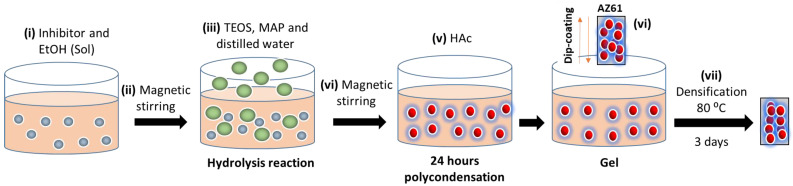
Sol–gel synthesis steps for coatings: (i) The corresponding inhibitor is dissolved in EtOH to form the sol. (ii) Homogenization of the sol by magnetic stirring. (iii) TEOS, MAP and distilled water are added dropwise where the hydrolysis process starts. (iv) Homogenization of the resulting solution. (v) Acetic acid (HAc) is added as catalyst where it is kept for 24 h under magnetic stirring. Then, (vi) the protective layer is deposited on the AZ61 alloy by dip-coating method. Finally, (vii) the densification treatment is carried out at 80 °C in air for 3 days.

The alkoxides tetraethylorthosilicate (TEOS, Sigma-Aldrich, Madrid, Spain, 99%) and 3-(trimethoxysilyl)propyl methacrylate (MAP/MEMO, Sigma-Aldrich, 98%) served as precursors for the sol–gel coatings. Ethanol (EtOH, Sigma-Aldrich, 96%) was utilized as a solvent. Distilled water was carefully added drop by drop for hydrolysis until the reaction reached completion. Finally, acetic acid was introduced as a catalyst to facilitate polycondensation. The molar ratios of the components were 1:1:4:8:0.01, respectively. Subsequently, environmentally friendly organic corrosion inhibitors were added to this formulation. Four different organic molecules that are environmentally friendly, non-toxic, inexpensive and contain S, N heteroatoms, O and/or OH groups and/or conjugated double bonds were selected: L-cysteine (CYS), N-acetyl-cysteine (N-A-CYS), curcumin (CUR), and methylene blue (MB) in a ratio of 0.8 wt.%. Figure 10 illustrates the chemical structure of each compound:

After mixing all the components, the sol was stirred with magnetic agitation for 24 h at 100 rpm to carry out the second stage of the sol–gel process, the polycondensation. Likewise, each solution includes the corresponding inhibitor, except for one called BLA, which does not include any inhibitor, and which will be subjected to the subsequent corrosion test. Afterward, the dip-coating method was used for the application of the coatings since it provides good surface finishes and achieves a uniform coating layer. The extraction on the protective solution was carried out at a constant speed of 8 mm/s. After coating has been carried out on all respective samples, they are heat-treated in a muffle at 80 °C to carry out the densification step. Figure 11 illustrates the described sol–gel synthesis steps:

## Figures and Tables

**Table 1:** Detailed description of the samples obtained.

Sample Name	Sol–Gel Coating	Corrosion Inhibitor Added	Sol’s Color	Sol’s pH	Corrosion Test Performed
A	NO	-	-	-	NO
REF	NO	-	-	-	YES
BLA	YES	-	Pink	2	YES
L-CYS	YES	L-cysteine	Pink	3–4	YES
N-A-CYS	YES	N-acetyl-cysteine	Red	2	YES
CUR	YES	Curcumin	Brown	2	YES
MB	YES	Methylene Blue	Blue	3	YES

**Table 2:** Calculated thicknesses of the coatings from the interference of reflection spectra.

Sample	BLA	L-CYS	N-A-CYS	CUR	MB
Thickness (nm)	195 ± 30	291 ± 40	281 ± 57	164 ± 30	268 ± 42

**Table 3:** EDX compositional analysis of the microstructure of the samples.

Sample	C (wt.%)	O (wt.%)	Mg (wt.%)	Al (wt.%)	Si (wt.%)	Cl (wt.%)
A-background	-	-	92.5 ± 1.7	7.5 ± 1.7	-	-
REF-background	-	57.3 ± 2.4	42.7 ± 2.4	-	-	-
REF-white goop	-	29.7 ± 2.7	66.7 ± 2.7	3.7 ± 1.2	-	-
REF-crystal	-	57.9 ± 2.4	42.1 ± 2.4	-	-	-
BLA-background	-	57.8 ± 2.5	39.5 ± 2.4	-	-	2.7 ± 0.8
BLA-triangle	-	57.4 ± 2.2	42.6 ± 2.2	-	-	-
L-CYS-background	-	23.0 ± 2.6	77.0 ± 2.6	-	-	-
L-CYS-smooth area	-	14.7 ± 2.5	78.3 ± 2.7	5.8 ± 1.4	1.2 ± 1.0	-
L-CYS-stick	-	51.7 ± 2.0	46.1 ± 2.0	2.3 ± 0.7	-	-
MB-background	-	38.1 ± 1.3	50.1 ± 1.2	2.2 ± 0.5	9.6 ± 0.7	-
CUR-smooth area	19.2 ± 2.4	17.7 ± 1.1	60.3 ± 2.0	2.8 ± 0.3	-	-
CUR-crust area	-	57.0 ± 1.1	43.0 ± 1.1	-	-	-
N-A-CYS-background	9.0 ± 2.1	43.1 ± 1.4	43.1 ± 1.3	1.1 ± 0.3	2.7 ± 0.3	1.0 ± 0.2

## Data Availability

The data presented in this study are openly available in article.
